# *Brucella suis* Vaccine Strain *2* Induces Endoplasmic Reticulum Stress that Affects Intracellular Replication in Goat Trophoblast Cells *In vitro*

**DOI:** 10.3389/fcimb.2016.00019

**Published:** 2016-02-09

**Authors:** Xiangguo Wang, Pengfei Lin, Yang Li, Caixia Xiang, Yanlong Yin, Zhi Chen, Yue Du, Dong Zhou, Yaping Jin, Aihua Wang

**Affiliations:** ^1^Key Laboratory of Animal Biotechnology of the Ministry of Agriculture, Northwest A&F UniversityYangling, China; ^2^College of Veterinary Medicine, Northwest A&F UniversityYangling, China

**Keywords:** goat trophoblast cells, *B.suis.S2*, endoplasmic reticulum stress, infection, apoptosis

## Abstract

*Brucella* has been reported to impair placental trophoblasts, a cellular target where *Brucella* efficiently replicates in association with the endoplasmic reticulum (ER), and ultimately trigger abortion in pregnant animals. However, the precise effects of *Brucella* on trophoblast cells remain unclear. Here, we describe the infection and replication of *Brucella suis* vaccine strain *2* (*B.suis.S2*) in goat trophoblast cells (GTCs) and the cellular and molecular responses induced *in vitro*. Our studies demonstrated that *B.suis.S2* was able to infect and proliferate to high titers, hamper the proliferation of GTCs and induce apoptosis due to ER stress. Tunicamycin (Tm), a pharmacological chaperone that strongly mounts ER stress-induced apoptosis, inhibited *B.suis.S2* replication in GTCs. In addition, 4 phenyl butyric acid (4-PBA), a pharmacological chaperone that alleviates ER stress-induced apoptosis, significantly enhanced *B.suis.S2* replication in GTCs. The Unfolded Protein Response (UPR) chaperone molecule GRP78 also promoted *B.suis.S2* proliferation in GTCs by inhibiting ER stress-induced apoptosis. We also discovered that the IRE1 pathway, but not the PERK or ATF6 pathway, was activated in the process. However, decreasing the expression of phosphoIRE1α and IRE1α proteins with Irestatin 9389 (IRE1 antagonist) in GTCs did not affect the proliferation of *B.suis.S2*. Although GTC implantation was not affected upon *B.suis.S2* infection, progesterone secretion was suppressed, and prolactin and estrogen secretion increased; these effects were accompanied by changes in the expression of genes encoding key steroidogenic enzymes. This study systematically explored the mechanisms of abortion in *Brucella* infection from the viewpoint of pathogen invasion, ER stress and reproductive endocrinology. Our findings may provide new insight for understanding the mechanisms involved in goat abortions caused by *Brucella* infection.

## Introduction

Brucellosis, which is caused by *Brucella* species, is one of the most common zoonoses worldwide (Schurig et al., 2002). Infection with *Brucella* results in a significant economic and health burden due to its high infectivity and chronic nature, as well as the difficulties in vaccine production (Taguchi et al., 2015). Abortion and infertility in adult animals are the main characteristics of brucellosis. *Brucella* vaccination is an important approach in the prevention and control of brucellosis (Nicoletti, 1990), and understanding the molecular mechanisms of *Brucella* pathogenesis and host response, such as intracellular trafficking and *Brucella* replication, is critical for vaccination production and curbing brucellosis. The bacterium resides within a *Brucella*-containing vacuole (BCV) after phagocytic uptake and entry into phagocytes or non-professional phagocytes (Celli et al., 2003). Along the endocytic pathway, BCVs undergo maturation and become endosomal BCVs (eBCVs) (Starr et al., 2012), and acidification of BCVs ensures the intracellular expression of genes that encode the VirB type IV secretion system (T4SS; Comerci et al., 2001; Celli et al., 2003, 2005). Some VirB effectors, including VecC, BspA, BspB, BspC, BspD, and BspF, have been used to modulate secretory pathway functions (de Barsy et al., 2011; Marchesini et al., 2011; Myeni et al., 2013). Host factors such as Sar1 (Kuge et al., 1994), Rab2 (de Barsy et al., 2011), glyceraldehyde-3-phosphate dehydrogenase (Fugier et al., 2009), and Yip1A (Taguchi et al., 2015) are required for the intracellular replication of *Brucella.*

*Brucella* can modulate phagosome interaction with the endoplasmic reticulum (ER), a large membrane-bound organelle involved in protein biosynthesis as well as lipid, carbohydrate and protein transport. Indeed, the ER plays an important role in cellular homeostasis by manipulating the processing and folding of membrane and secretory proteins (Pluquet et al., 2015). However, when protein processing and folding requirements exceed the capacity of the ER, unfolded proteins accumulate, evoking ER stress and inducing the unfolded protein response (UPR), which involves inositol-requiring enzyme 1 (IRE1), double-stranded RNA-dependent protein kinase P (PKR)-like ER kinase (PERK) and activating transcription factor 6 (ATF6) (Schröder and Kaufman, 2005). Many studies have confirmed that induction of the UPR pathway following *B. abortus* and *B. melitensis* infection promotes the intracellular growth of these bacteria (Qin et al., 2008; Smith et al., 2013; Taguchi et al., 2015), especially the IRE1α pathway (Qin et al., 2008; Taguchi et al., 2015). However, when UPR fails to manage misfolded and unfolded proteins, cellular apoptosis is induced due to persistent or excessive ER stress (Walter and Ron, 2011). Survival and replication inside macrophages is critical for the establishment of chronic *Brucella* infection. Virulent smooth *Brucella* strains, such as *B. abortus* strain 2308 and smooth *B. suis* (Gross et al., 2000; Tolomeo et al., 2003; He et al., 2006), inhibit programmed macrophage cell death and replicate inside macrophages. In contrast, rough *B. abortus* strains, such as RB51 and RA1 (Chen and He, 2009), induce macrophage cell death through a caspase2-dependent pathway. Two death signal pathways control cell apoptosis: the mitochondria C pathway and the death receptor pathway. Caspases are crucial components in the execution of apoptosis. Caspase-8 is the initiator caspase in the death receptor pathway, whereas caspase-9 is the initiator caspase in the mitochondrial pathway; caspase-3 is the key executioner caspase in all apoptosis pathways (Li and Yuan, 2008; Kantari and Walczak, 2011). ER stress-mediated apoptosis is a new apoptosis signaling pathway (Gorman et al., 2012), and the specific mechanism of ER stress apoptosis in *Brucella* infections is attracting much research interest.

Trophoblasts are cellular targets where *Brucella* efficiently replicates in association with the ER. Recent studies have confirmed that ER stress is closely related to placental functions, such as placental development, fetal growth restriction (Yang et al., 2015), progesterone secretion, and steroidogenic enzyme expression (Park et al., 2014). Altering the features of placental trophoblast cells will result in a variety of complications during pregnancy. Under normal circumstances, goat pregnancy is typically maintained by a high concentration of progesterone, whereas the concentration of estrogen is very low. Before delivery, progesterone secretion decreases, and estrogen secretion increases to occupy a dominant position. Such an increase in estrogen or a reduced progesterone to estrogen ratio promotes the secretion of prolactin and initiates lactation (Cole and Cupps, 1969). In addition, estrogen promotes the expression of PR; however, progesterone inhibits the expression of PR (Devillers et al., 2006), resulting in the maximum receptivity capacity of the endometrium during embryo implantation. Finally, the decrease in PR stimulates endometrial function differentiation and the production of secretory proteins (Devillers et al., 2006). *Brucella* strains can infect and proliferate within placental trophoblasts during pregnancy in an infected host (Samartino and Enright, 1993), which has significant pathological consequences, such as abortion and infertility (Kurdoglu et al., 2010). It is known that *B. abortus* replication occurs in the rough endoplasmic reticulum in infected caprine trophoblasts (Anderson and Cheville, 1986); however, the specific mechanism underlying abortion in *Brucella* infections remains unknown.

Here, we investigate the mechanisms of ER stress induced by *B.suis.S2* infection and replication in GTCs. Our results showed that GTC apoptosis and growth retardation were induced by *B.suis.S2* replication under ER stress. Changes in ER stress in GTCs influenced the proliferation of *B.suis.S2*, and both the endocrine balance of trophoblast cells and endometrial receptivity were defective during *B.suis.S2* infection. Our findings may provide new insight for understanding the mechanisms involved in goat abortions caused by *Brucella* infections.

## Materials and methods

### Bacterial strain preparation and cultivation

*Brucella suis* vaccine strain *2* (*B.suis.S2*) cells were cultured in 50 ml of tryptic soy broth (TSB) medium with constant agitation at 37°C. The bacteria were collected by centrifugation at 6000 × g for 20 min at 4°C and washed three times with 15 ml of phosphate-buffered saline (PBS). The numbers of *B.suis.S2* cells were counted by plating on tryptic soy agar (TSA). pFPV-mCherry was a gift from Olivia Steele-Mortimer (Addgene plasmid # 20956), and the plasmid was transformed into *B.suis.S2* to obtain *B.suis.S2*-mCherry (red). All procedures involving live *B.suis.S2* cells were performed in a biosafety level 3 facility.

### *B.suis.S2* infection assays

**Figure 1:**
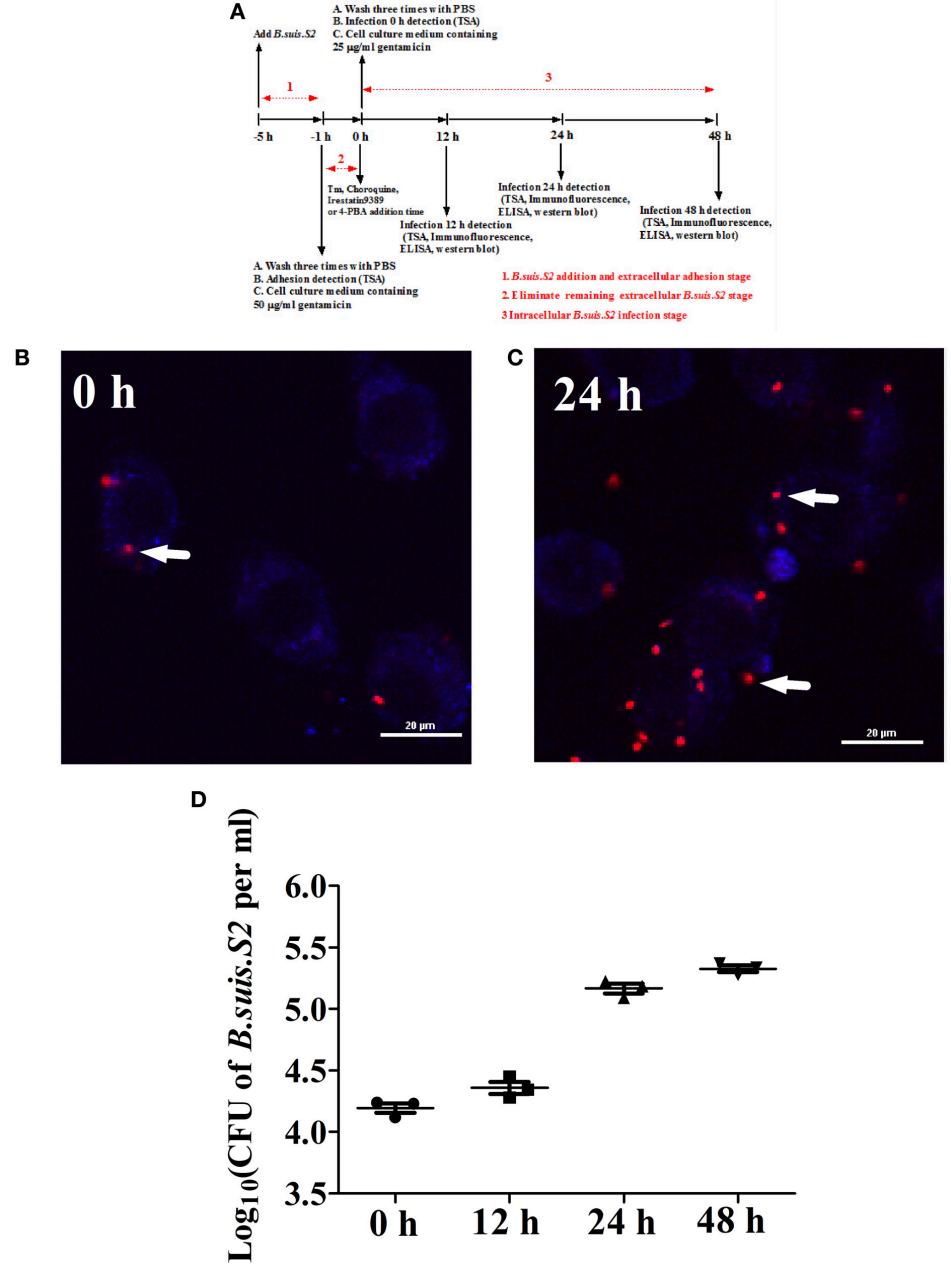
**Infection and proliferation of *B.suis.S2* in GTCs. (A)** Schematic of *B.suis.S2* infection. From −5 to −1 h is the *B.suis.S2* addition and extracellular adhesion stage; −1 to 0 h is the stage in which extracellular *B.suis.S2* is eliminated by adding 50 μg/ml gentamicin to the cell culture medium; 0–48 h is the intracellular *B.suis.S2* infection stage; 0 h is the time of Tm (ER stress activator), 4-PBA (ER stress antagonist), Irestatin 9389 (IRE1αantagonist), or chloroquine (autophagy antagonist) addition. “−” represents pretreatment before the time point (0 h). **(B,C)** *B.suis.S2*-mCherry (MOI = 100:1)-infected GTCs at 0 h **(B)** and 24 h **(C)** (bar = 20 μm). The white arrows indicate *B.suis.S2*-mCherry in the GTCs. The images in B and C are representative of 3–4 independent experiments. **(D)** Intracellular multiplication of *B.suis.S2* (MOI = 100:1). CFU numbers were determined after the lysis of infected cells at the indicated times post-infection by TSA plate counting. CFU numbers are shown on a log_10_ scale. All data represent the means ± standard deviations from 3 independent experiments.

Goat trophoblast cells (GTCs) were immortalized by transfection with human telomerase reverse transcriptase (hTERT; Dong et al., 2013); these cells were provided by professor Dewen Tong (Northwest A&F University, Yangling, Shaanxi, China). A standard gentamicin protection assay was carried out to determine the number of intracellular *B.suis.S2* bacteria. GTCs were seeded in six-well plates (5 × 10^5^ cells per well) and infected with *B.suis.S2* at 100:1 MOI. Multiwell plates were placed at 37°C in 5% CO_2_. After 4 h of incubation, the GTCs were washed three times with PBS and further cultured with cell culture medium containing 50 μg/ml gentamicin to eliminate *B.suis.S2* cells adhering to the GTCs and in the culture medium. After 1 h, the GTCs were washed three times with PBS and further cultured with cell culture medium containing 25 μg/ml gentamicin. This time was considered to be the beginning of the intracellular growth of *B.suis.S2* within GTCs and the time point of treatment with Tm (ER stress activator), 4-PBA (ER stress antagonist), Irestatin9389 (IRE1α antagonist), or chloroquine (autophagy antagonist). The cells and supernatants were collected, and relevant experiments were performed at specific times (−1, 12, 24, or 48 h). A schematic of the *B.suis.S2* infection assays is presented in Figure 1A. The process of *B.suis.S2*-mCherry infection of GTCs was observed under a Nikon A1R si confocal microscope system, and the numbers of invasive *B.suis.S2* bacteria were determined. GTCs were treated with 0.1 ml of 0.1% Triton X-100 in PBS for 5 min at 37°C, and the lysates were diluted in PBS and plated onto TSA to determine the colony-forming units (CFU; Posadas et al., 2012).

### GTC cultivation and screening of GRP78 shRNA and over-expressing stable transfection GTC cell lines

GTCs were cultured in DMEM/F12 (Invitrogen) at 1 × 10^5^ cells per well supplemented with 10% FCS (Gibco) streptomycin in a 5% CO_2_ atmosphere at 37°C for 24 h. Four types of recombinant lentiviral supernatants and control lentiviral supernatants at an MOI of 20 were diluted with culture medium containing 8 μg/ml of polybrene and incubated for 8 h. The old medium containing the virus was then removed and replaced with fresh culture medium. After 48 h, puromycin (5 μg/ml) was added to the medium to eliminate the GTCs uninfected by the lentivirus. The transfected GTCs were harvested after 72 h, and total proteins were isolated to detect GPR78 gene expression. The GTCs were then infected with *B.suis.S2* at an MOI of 100; the GTCs and supernatants were harvested, and relevant experiments were performed at specific times (12, 24, or 48 h).

### Cell counting, cell cycle assay, and flow cytometry

GTCs (2 × 10^4^) were cultured in 24-well plates at the initial time in the control and in the *B.suis.S2*-infected group. *B.suis.S2*-infected GTCs and non-infected GTCs were separately trypsinized and counted by cell count plating at 24 h after *B.suis.S2* infection. Cell cycle distribution was analyzed using flow cytometry (FCM). Briefly, GTCs were trypsinized at 24 h after *B.suis.S2* infection, washed three times with PBS, and fixed with 70% ethanol. Fixed GTCs were washed three times with PBS and incubated with 20 mg/ml RNase for 30 min before staining with propidium iodide (PI; Sigma, CA). The GTCs were then analyzed by FCM (Beckman Coulter Cytomics Altra). To determine the percentage of apoptotic GTCs, the above GTCs were quantified using an Annexin V-FITC Apoptosis Detection Kit (KGA107, Nanjing Keygen Biotech Co., Ltd.). The GTCs were washed three times with cold PBS using centrifugation, and their density was adjusted to 1 × 10^6^ cells per milliliter. The GTCs were then resuspended in 500 μl binding buffer; 5 μl annexin V-FITC and 5 μl PI were added and incubated for 20 min at 4°C in the dark. Detection by flow cytometry (EPICS Altra, Beckman Coulter Cytomics Altra) was performed within 1 h.

### Immunofluorescent staining

GTCs were cultured in 24-well plates and infected with *B.suis.S2* or *B.suis.S2*-mCherry for 24 h. Immunofluorescent staining of caspase-3, GRP78, CHOP, phosphoIRE1α, IRE1α, and LC3 was performed. The GTCs were fixed in 4% paraformaldehyde for 30 min and then permeabilized for 15 min with 0.1% Triton X-100 in PBS, subsequently blocked for 1 h with 5% BSA in PBS at room temperature, and co-incubated with anti-caspase-3 (Santa Cruz, 1:50 dilution), anti-CHOP (Santa Cruz, 1:50 dilution), anti-GRP78 (Santa Cruz, 1:50 dilution), anti-phosphoIRE1α (Abcam, 1:500 dilution), anti-IRE1α (Santa Cruz, 1:50 dilution), or anti-LC3 (Sigma, 1:500 dilution) antibodies at 37°C for 2 h. After washing and incubation with an anti-rabbit secondary antibody (for CHOP, phosphoIRE1α, IRE1α, LC3, and caspase-3) (Invitrogen, A21206; 1:500 dilution) or an anti-goat secondary antibody (for GRP78) (Invitrogen, A21432; 1:500 dilution) at 37°C for 1 h, the nuclei were stained with 4′, 6-diamidino-2-phenylindole (DAPI) for 3–5 min. The fluorescent signals were examined under a Nikon A1R si confocal microscope system.

### Real-time reverse transcription-polymerase chain reaction (real-time RT-PCR)

Total RNA of *B.suis.S2*-infected GTCs was extracted using TRIzol (Invitrogen, Inc., Carlsbad, CA, USA). cDNA was synthesized using PrimeScript™ RT Reagent Kit (TaKaRa Bio, Inc., Dalian, China) according to the manufacturer's protocols. Real-time PCR was subsequently performed using an ABI 7500 Sequencing Detection System and SYBR Premix Ex Taq™. The GenBank accession numbers and primer sequences of *matrix metallopeptidase 2* (*MMP2*), *matrix metallopeptidase 9 (MMP9*), *PERK, IRE1, ATF6, CYP19A1, CYP17A1, StAR, HSD3B*, and *GAPDH* are summarized in Table 1. All reactions were performed in at least three independent experiments, and the calculated number of copies of the target genes was normalized to the number of *GAPDH* mRNA copies in the same sample.

**Table 1:** **Sequences of the primers used for qRT-PCR**.

**Target gene**	**GenBank accession no**.	**Primer sequence**	**Product size (bp)**	**Annealing temperature (°C)**
MMP2	NC_022310.1	AF:5′-CCTGCAAGTTCCCGTTCC-3′	84	60
		AR: 5′-ACACCAGCGGTAGCCATCC-3′		
MMP9	NC_022305.1	AF:5′-GGTGGACTATGTGGGCTACG-3′	127	60
		AR:5′-GACTGGCTCATTCCCTACTGG-3′		
PERK	XM_005686691.1	AF:5′-CCCCATCCGCTACTGAACG-3′	151	60
		AR:5′-GGGCTGCTGGAGTGTCTTG-3′		
IRE1	XM_005694366.1	AF:5′-ACTCCCTCAACATCGTTCACAG-3′	208	60
		AR:5′-CTCCTTGCAGTCTTCGCTCA-3′		
ATF6	AY942654	AF:5′-AACCAGTCCTTGCTGTTGCT-3′	224	60
		AR:5′-CTTCTTCTTGCGGGACTGAC-3′		
StAR	XM_005698829.1	AF:5′-AGGCCATGGGCGAGTGGAAC	145	60
		AF:5′-GTACAGCGCACGCTCACAAA-3′		
HSD3B	NM_001285716.1	AF:5′-TCCACACCAGCACCATAGAG-3′	143	60
		AF:5′-TTCCAGCACAGCCTTCTCG-3′		
CYP17A1	XM_005698413.1	AF:5′-GGCCCAAGACCAAGCACTC-3′	161	60
		AF:5′-GGAACCCAAACGAAAGGAATAG-3′		
CYP19A1	NM_001285747.1	AF:5′-ATCTGTGCTGATTCCATCAC-3′	118	60
		AF:5′-GGATGTTAGAGGTGTCCAGCA-3′		
GAPDH	XM_005680968.1	AF:5′-GGCGCCAAGAGGGTCAT-3′	100	60
		AF:5′-GTGGTTCACGCCCATCACA-3′		

All primers were synthesized by Sangon Biotech Co., Ltd. (Shanghai, China).

### Western blot analysis

*B.suis.S2*-infected GTCs were harvested and lysed on ice for 30–45 min in lysis buffer. The supernatant was then collected in a new tube after centrifugation for 15 min at 14,000 rpm at 4°C. The protein concentration was calculated by the BCA assay. Total cellular protein (40 μg) was electrophoresed on a 12% sodium dodecyl sulfate polyacrylamide gradient (SDS-PAGE) gel and electro-transferred onto PVDF membranes. The membranes were blocked for 1 h with 5–10% non-fat milk in Tris-buffered saline containing 0.5% Tween-X-100 (TBST) at room temperature and then incubated overnight at 4°C in blocking solution containing anti-GRP78 (Santa Cruz, 1:100 dilution), anti-CHOP (Santa Cruz, 1:100 dilution), anti-caspase-3 (Santa Cruz, 1:100 dilution), anti-IRE1α (Santa Cruz, 1:200 dilution), anti-phosphoIRE1α (Abcam, 1:1,000 dilution), anti-LC3 (Sigma, 1:1,000 dilution), anti-MAPK8 (Takara, 1:100 dilution), anti-caspase-8 (Takara, 1:100 dilution), anti-caspase-9 (Takara, 1:100 dilution), anti-PR (Santa Cruz, 1:100 dilution), anti-EαR (Abcam, 1:500 dilution), or anti-β-actin (Tianjin Sungene Biotech Co., Ltd, 1:2000 dilution) primary antibodies. The next day, the membranes were washed with TBST and incubated with the corresponding secondary antibody conjugated to HRP (1:2000; Zhongshan Golden Bridge Biotechnology, Nanjing, China) for 1 h at room temperature. Finally, the immunoreactive bands were visualized using the Gel Image System (Tannon, Biotech, Shanghai, China) and then digitized with Quantity One software.

### Migration and invasion assay

*B.suis.S2*-infected GTC migration and invasion were assessed using a 24-well plate BD Bio-Coat Matrigel Invasion Chamber (BD Biosciences, Bedford, MA). The inserts contained polyethylene terephthalate membranes (8 μm pore size) coated with (invasion) or without (migration) a thin layer of Matrigel and a reconstituted basement membrane that prevented non-invasive cells from migrating through the pores; invasive cells were able to invade and migrate through the Matrigel-coated membrane. The experiments were performed according to the manufacturer's instructions. The GTCs (2 × 10^4^) were trypsinized, counted, and resuspended in serum-free medium; 500 μl of medium containing 10% FBS (the chemoattractant) was added to each well, and 200 μl of cell suspension was loaded into the upper well. The plate was incubated for 24 h at 37°C in a 5% CO_2_ atmosphere, after which cotton swabs were used to remove non-invading cells from the upper surface of the filter. The GTCs on the lower surface of the Matrigel (invasion) or basement membrane (migration) were fixed for 30 min in 4% paraformaldehyde, washed three times with PBS, stained with crystal violet, and observed using a Nikon invert optical microscope (Nikon Eclipse 80i; Nikon, Tokyo, Japan).

### Determination of prolactin, estrogen, and progesterone levels

GTCs were infected with *B.suis.S2* (MOI = 100) as described above. Supernatants were harvested at specific times and centrifuged at 2500 × g for 5 min, filtered through a 0.22 μm filtration membrane, aliquoted into small volumes and stored at −80°C until use. The concentrations of prolactin, estrogen, and progesterone in the culture media were assayed using ELISA kits (BD Bioscience, San Jose, CA, USA) according to the manufacturer's recommendations. The optical densities at 450 nm of each well were determined using a micro-plate reader (Model 680, Bio-Rad, Hercules, CA, USA).

### Statistical analysis

The experimental results are presented as the means ± standard deviations, as derived from at least three independent experiments. Data were analyzed with one-way ANOVA followed by Fisher's least significant difference test (Fisher LSD) and the Independent-Samples *T*-test using the SPSS (Statistical Package for the Social Sciences) software (Version 16.0; SPSS, Inc., Chicago, IL). Differences were considered significant when *P* < 0.05.

## Results

### *B.suis.s2* infects and replicates in GTCs

Our results from the gentamicin protection assay confirmed that *B.suis.S2* was able to infect GTCs cultured *in vitro*. *B.suis.S2*-mCherry was primarily found within the cells after the *B.suis.S2* bacteria that had adhered to the GTCs and in the culture medium were killed by gentamicin (Figure 1B). Approximately 99% of GTCs were infected with *B.suis.S2*-mCherry at an MOI of 100 at 24 h post-infection (Figure S1). In addition, the intracellular replication of *B.suis.S2* occurred in a time-dependent manner (Figure 1C), and the bacterial numbers rapidly increased after 12 h (Figure 1D).

### Effect of *B.suis.S2* on GTC viability

**Figure 2:**
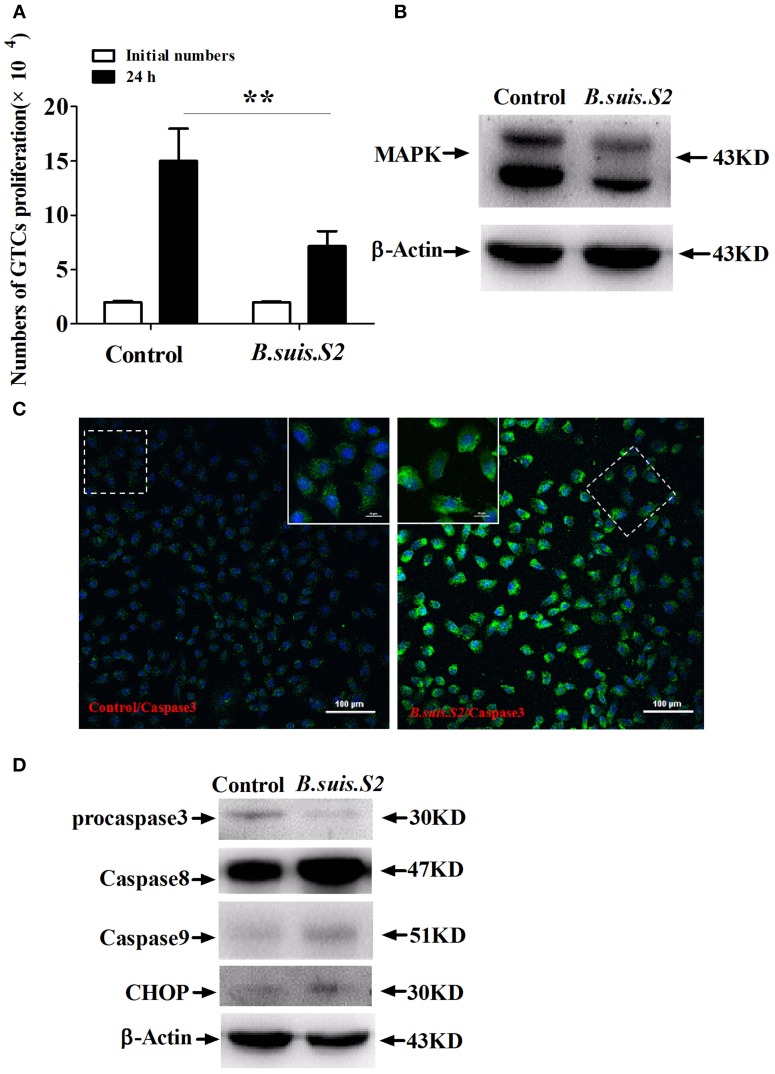
**Cell proliferation and apoptosis of *B.suis.S2*-infected GTCs. (A)** The proliferation numbers of GTCs infected by *B.suis.S2* (MOI = 100:1) were determined by cell counting. All data represent the means ± standard deviations from 4 independent experiments. ^**^P < 0.01 vs. non-infected cells. **(B)** GTCs were infected with 100 MOI of *B.suis.S2* for 24 h, lysed and subjected to Western blot analysis to detect MAPK8 protein expression. The image shown is representative of 3–4 independent experiments. **(C)** Confocal microscope images of total caspase-3 protein expression in *B.suis.S2*-infected GTCs at 24 h post-infection. The data shown are representative of 4 independent experiments. **(D)** GTCs were infected with 100 MOI of *B.suis.S2* for 24 h, lysed and subjected to Western blot analysis to detect the expression of apoptosis-related genespro-caspase-3, caspase-8, caspase-9, and CHOP proteins. The data shown are representative of 4–5 independent experiments.

To clarify the viability of GTCs infected with *B.suis.S2*, we assessed the effect of *B.suis.S2* on proliferation and apoptosis. Our results showed that *B.suis.S2* infection inhibited GTC proliferation at 24 h post-infection compared to the uninfected group (Figure 2A). To confirm these results, we examined the mitotic cycle of GTCs after 24 h of *B.suis.S2* infection using flow cytometry. At 24 h post-infection, 61.0 ± 3.39% of the GTCs were in S phase, and 8.43 ± 0.87% of the GTCs were in G2 phase; in contrast, in the uninfected group, 33.1 ± 1.54% of the GTCs were in S phase, and 36.2 ± 2.37% of the GTCs were in G2 phase (Table 2). Overall the mitotic cycle of GTCs infected with *B.suis.S2* was stalled in S phase. Western blotting showed that mitogen-activated protein kinase 8 (MAPK8) expression significantly decreased at 24 h after *B.suis.S2* infection compared to the uninfected group (Figure 2B).

**Table 2:** **Results of flow cytometry assays for cell cycle distribution in *B.suis.S2*-infected GTCS**.

**Group**	**G1 stage (%)**	**G2 stage (%)**	**S stage (%)**
Non-infected	30.50 ± 1.87	36.20 ± 2.37	33.10 ± 1.54
*B.suis.S2*-infected	30.70 ± 2.02	8.43 ± 0.87^**^	61.00 ± 3.39^**^

Data represent the means ± standard deviations from 4 replicates. The asterisks (

** ) represent significant differences (P < 0.01) of the cell cycle distribution in GTCs infected by B.suis.S2 compared to that in uninfected GTCs.

Because *B.suis.S2* infection reduced cell viability, we measured apoptosis in *B.suis.S2*-infected GTCs using flow cytometry, caspase-3 expression assays and Western blotting after 24 h of *B.suis.S2* infection. *B.suis.S2*-induced apoptosis was quantified by flow cytometry in combination with Annexin V/PI double staining. When the cells were infected with *B.suis.S2* for 24 h, the average proportion of Annexin V-positive cells (total number of apoptotic cells) increased significantly, reaching approximately 20.2 ± 2.29%. However, the average proportion of total apoptotic cells to non-infected cells was only approximately 7.77 ± 0.56% (Table 3). Total caspase-3 protein expression was significantly increased after 24 h of *B.suis.S2* infection compared to the non-infected group (Figure 2C). Western blot analysis showed that full-length procaspase-3 decreased in the *B.suis.S2* infection group at 24 h post-infection, whereas caspase-9 and caspase-8 increased in the *B.suis.S2* infection group at 24 h post-infection. CHOP, a marker of ER stress that induces apoptosis, was also induced in the *B.suis.S2* infection group at 24 h post-infection (Figure 2D).

**Table 3:** **Results of Annexin V-FITC/PI staining for cell apoptosis after *B.suis.S2* infection**.

**Group**	**Progressed apoptotic cells (%)**	**Early apoptotic cells (%)**	**Survival cells (%)**
Non-infected	4.41 ± 0.33	3.29 ± 0.23	92.30 ± 1.70
*B.suis.S2*-infected	13.22 ± 1.39^*^	6.98 ± 0.90^*^	79.80 ± 2.01^*^

Data represent the means ± standard deviations from 4–5 replicates. The asterisk (

* ) represents significant differences (P < 0.05) in cell apoptosis in GTCs infected by B.suis.S2 compared to that in uninfected GTCs.

### Detection of GRP78 and CHOP expression in GTCs infected with *B.suis.S2*

**Figure 3:**
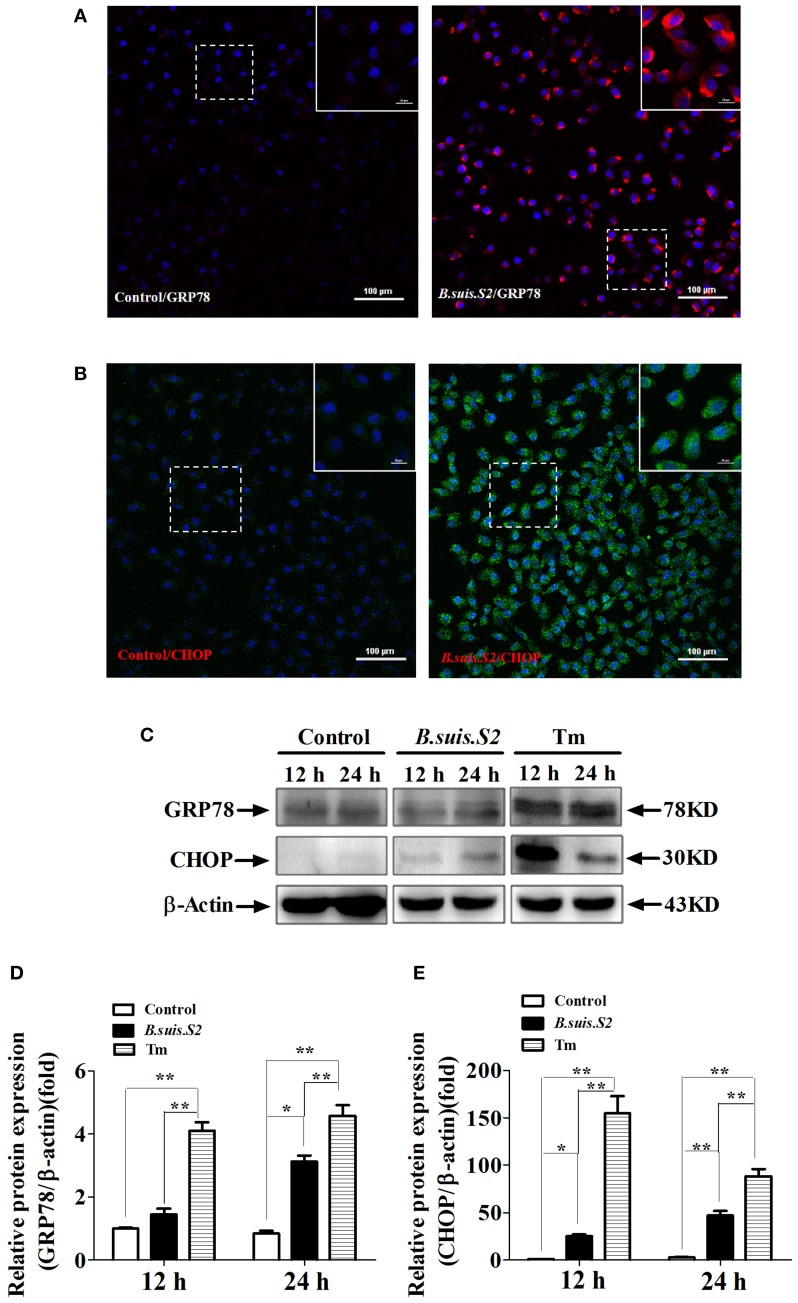
**ER stress induced in *B.suis.S2*-infected GTCs**. Confocal microscope images of GRP78 **(A)** and CHOP **(B)** protein expression in *B.suis.S2*-infected GTCs (MOI = 100:1) at 24 h. The data shown are representative of 4–5 independent experiments. **(C)** GTCs were infected with 100 MOI of *B.suis.S2* for 12 and 24 h. The Tm-treated group was used as a positive control, lysed and subjected to Western blot analysis to detect GRP78 and CHOP protein expression. The data shown are representative of 5 independent experiments **(D,E)**. Quantification of band intensities from 5 independent results was determined by densitometric analysis. Data represent the mean ± standard deviations from 5 independent experiments (^*^P < 0.05, ^**^P < 0.01).

UPR associated with the physiological response is used to monitor the proliferation of intracellular pathogens (Cho et al., 2013). To more directly examine ER stress after *B.suis.S2* infection in GTCs, we examined the expression of GRP78 and CHOP proteins in *B.suis.S2*-infected GTCs. GRP78 expression was enhanced in more than 99% of GTCs at 24 h after *B.suis.S2* infection (Figure 3A), in agreement with the *B.suis.S2* infection rate. Additionally, CHOP protein expression was basically similar to GRP78 protein expression at 24 h in *B.suis.S2*-infected GTCs (Figure 3B). According to our Western blot results, GRP78 protein expression was more strongly induced in the *B.suis.S2* infection group than in the uninfected group at 24 h post-infection but was weaker than that induced by Tm at 12 and 24 h post-infection (Figures 3C,D). CHOP protein expression was also more strongly induced in the *B.suis.S2* infection group than in the uninfected group and was weaker than that induced by Tm at 12 and 24 h (Figures 3C,E).

### Changing ER stress affects *B.suis.S2* intracellular growth in GTCs *in vitro*

**Figure 4:**
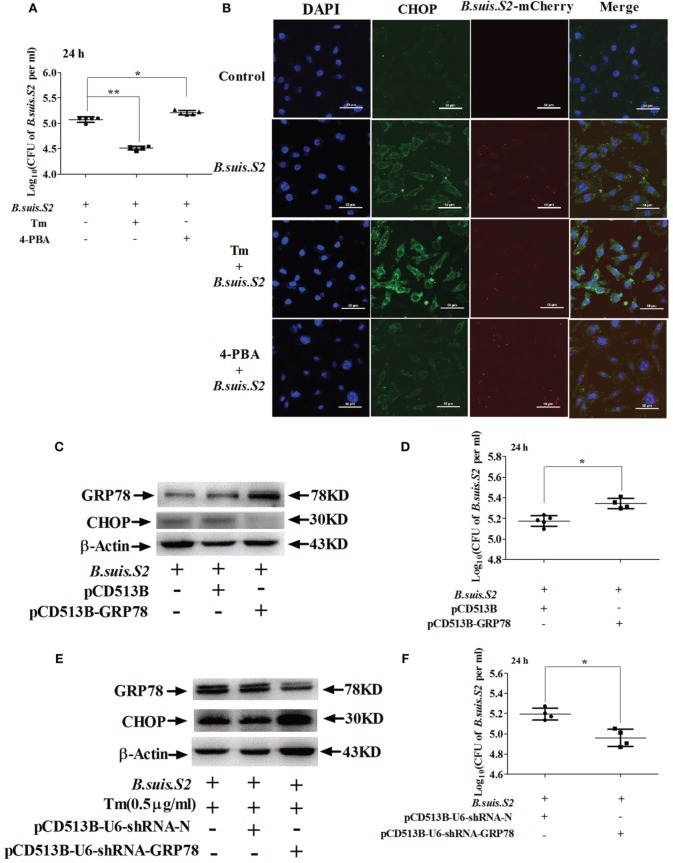
**Manipulating ER stress in GTCs affects *B.suis.S2* replication. (A)** GTCs were infected with 100 MOI *B.suis.S2* at −5 h. Then, 0.5 μg/mL Tm or 1 μM 4-PBA was added at 0 h. Cells were lysed after 24 h of *B.suis.S2* infection. CFUs were determined by transfer to dilution plates. CFU numbers are shown on a log_10_ scale. Data represent the mean ± standard deviations from 5 independent experiments. ^*^P < 0.05, ^**^P < 0.01 vs. *B.suis.S2*-infected cells. **(B)** Confocal microscope images of CHOP protein expression in GTCs infected with *B.suis.S2* only or plus 0.5 μg/mL Tm or 1 μM 4-PBA at 24 h. The data shown are a representative of 4–5 independent experiments. **(C–F)** The ER stress chaperone GRP78 promotes *B.suis.S2* replication. **(C)** Over-expression of GRP78 in GTCs by pCD513B-GRP78 lentivirus transduction was detected with Western blot analysis. pCD513B was used as a control. Data shown are representative of 5 independent experiments. **(D)** Intracellular multiplication of *B.suis.S2* CFU numbers (MOI = 100:1) was determined after the lysis of infected cells (GRP78 overexpression) at the indicated times post-infection using TSA plate counting. CFU numbers are shown on a log_10_ scale. Data represent the mean ± standard deviations from 4–5 independent experiments. ^*^P < 0.05 versus the control. **(E)** Effective inhibition of GRP78 expression in 0.5 μg/ml Tm-treated GTCs by pCD513B-U6-shRNA-GRP78 lentivirus transduction was detected by Western blot analysis. pCD513B-U6-shRNA-N was used as a control. Data shown are representative of 5 independent experiments. **(F)** Intracellular multiplication of *B.suis.S2* in CFUs (MOI = 100:1) was determined after the lysis of infected cells (GRP78 interference) at the indicated times post-infection using TSA plate counting. CFU numbers are shown on a log_10_ scale. Data represent the mean ± standard deviations from 4 independent experiments (^*^P < 0.05).

As decreasing ER stress with TUDCA reduces *B. melitensis* proliferation in RAW 264.7 cells (Smith et al., 2013), we explored whether ER stress altered by Tm or 4-PBA affects the intracellular growth of *B.suis.S2* in GTCs. Increasing ER stress with 0.5 μg/ml Tm (Figures S2A,C) significantly increased CHOP protein expression and inhibited the proliferation of *B.suis.S2* at 24 h post-infection compared to untreated infected GTCs. Compared with Tm administration, decreasing ER stress with 1 μM 4-PBA (Figures S2B,C) significantly inhibited CHOP protein expression and promoted *B.suis.S2* proliferation at 24 h post-infection compared to untreated infected GTCs (Figures 4A,B). This observation is consistent with other reports that *Brucella* proliferation in host cells is affected by apoptosis in infected cells (Chen and He, 2009).

Moderate induction of BiP/GRP78 protects host cells from prolonged ER stress, augmenting UPR-mediated signaling and subsequent host cell apoptosis (Shima et al., 2015). We therefore screened stably transfected GRP78 interference and over-expressing GTCs using lentivirus packaging technology. The screened GTCs met the requirements of the subsequent experiments, as confirmed by Western blotting (Figures 4C,E). Increased GRP78 expression inhibited expression of CHOP and promoted the proliferation of *B.suis.S2* at 24 h post-infection (Figure 4D), whereas inhibiting GRP78 increased CHOP protein expression and inhibited the proliferation of *B.suis.S2* at 24 h post-infection (Figure 4F).

### Infection with *B.suis.S2* activates the IRE1 pathway of UPR

**Figure 5:**
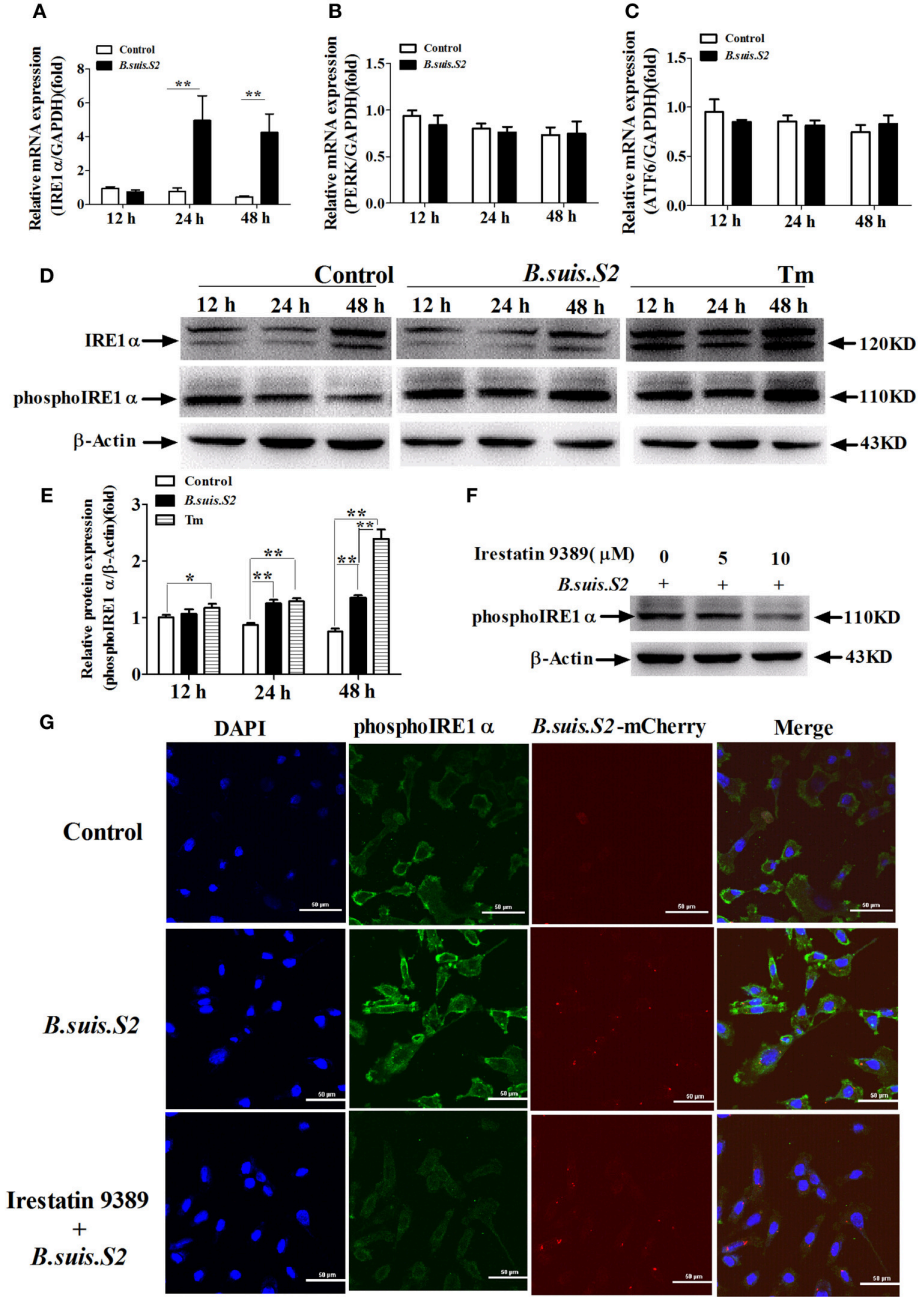
**The UPR pathway is induced after *B.suis.S2* infection**. The mRNA expression levels of *IRE1*α **(A)**, *ATF6* **(B)**, and *PERK* **(C)** are shown for the control and *B.suis.S2*-infected cells (MOI = 100:1) at 12, 24, and 48 h. Real-time PCR results from 5–6 separate experiments are shown (the data are corrected for expression of the housekeeping gene *GAPDH*, mean ± standard deviations), ^**^P < 0.01 versus non-infected cells. **(D)** GTCs were infected with 100 MOI of *B.suis.S2* for 12, 24, and 48 h, lysed and subjected to Western blot analysis to detect IRE1α and phosphoIRE1α expression. The data shown are representative of 4–5 independent experiments. **(E)** Quantification of phosphoIRE1α band intensities from three independent results was determined by densitometric analysis. Data represent the mean ± standard deviations from 4 independent experiments (^*^P < 0.05, ^**^P < 0.01). **(F)** GTCs were infected with 100 MOI of *B.suis.S2* alone or with Irestatin 9389 (5 or 10 μM) for 24 h, lysed and subjected to Western blot analysis to detect phosphoIRE1α protein expression. The data shown are representative of 5 independent experiments. **(G)** Confocal microscope images of phosphoIRE1α expression in *B.suis.S2*-infected GTCs with or without 10 μM Irestatin 9389 at 24 h. The data shown are representative of 4 independent experiments.

To investigate UPR induction during *B.suis.S2* infection, GTCs were infected (or not) with *B.suis.S2*, and the activation of three UPR sensors (*IRE1*α*, PERK*, and *ATF6*) was analyzed by qRT-PCR. The mRNA expression of *IRE1*α increased after *B.suis.S2* infection at 24 and 48 h post-infection compared to the uninfected group (Figure 5A). In contrast, the expression of *PERK* and *ATF6* was fairly constant over time in both control and infected cells (Figures 5B,C). The level of phosphoIRE1α correlated with a marked increase in *IRE1*α mRNA at 24 and 48 h post-infection, yet that induced after *B.suis.S2* infection was lower than that induced by Tm administration at 48 h (Figures 5D,E). Decreasing phosphoIRE1α and IRE1α protein expression with 10 μM Irestatin 9389 in GTCs did not affect the numbers of *B.suis.S2* bacteria at 24 h post-infection (Figures 5F,G, Figure S3). IRE1α is required for autophagy and functions together with the autophagy-related proteins Atg9 and WIPI1 (Ogata et al., 2006). In our study, expression of LC3 protein was more strongly induced in the *B.suis.S2* infection group than in the uninfected group at 12, 24, and 48 h post-infection (Figure S4A). Furthermore, decreasing LC3 protein expression in GTCs with 10 μM chloroquine significantly decreased the numbers of *B.suis.S2* bacteria at 24 h post-infection (Figures S4B,C).

### *B.suis.S2* did not affect GTC migration and invasion capacities

**Figure 6:**
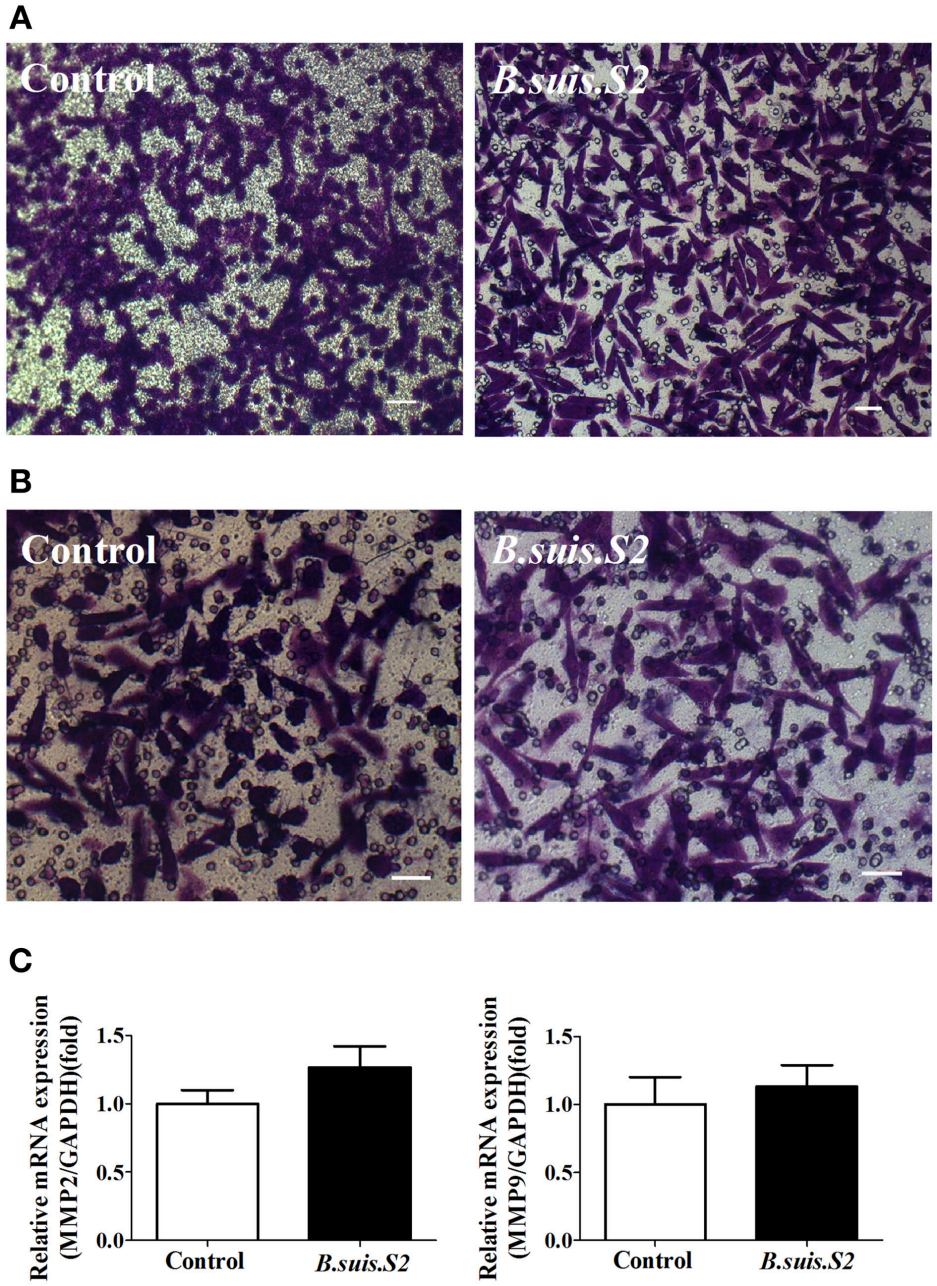
**Effect of *B.suis.S2* on the functions of GTCs. (A)** The migration ability of *B.suis.S2* (MOI = 100:1)-infected GTCs was measured using a Transwell chamber migration assay. Crystal violet was used to stain the migrated cells (bar = 10 μm). The data shown are representative of 4 independent experiments. **(B)** The invasion ability of *B.suis.S2* (MOI = 100:1)-infected GTCs was measured using a Matrigel invasion chamber assay. Crystal violet was used to stain the invaded cells (bar = 20 μm). The data shown are representative of 4 independent experiments. **(C)** The mRNA expression levels of *MMP2* and *MMP9* are shown for the control at 24 h. The results of real-time PCR (the data are corrected for expression of the housekeeping gene GAPDH; mean ±standard deviations from 5–6 independent experiments, *P* < 0.05).

As trophoblasts have important implantation functions throughout pregnancy, we assayed the ability of *B.suis.S2*-infected GTCs to migrate and invade a Matrigel basement membrane matrix using a standard *in vitro* assay. Microscopy-facilitated counting revealed no significant difference in migration ability with and without *B.suis.S2* infection (Figure 6A), Similarly, invasion ability was also not significantly different (Figure 6B). Furthermore, mRNA expression of both *MMP2* and *MMP9* in the *B.suis.S2* infection group was not significantly different from the non-infected group (Figure 6C).

### *B.suis.S2* disturbed the balance of hormone secretion by GTCs

**Figure 7:**
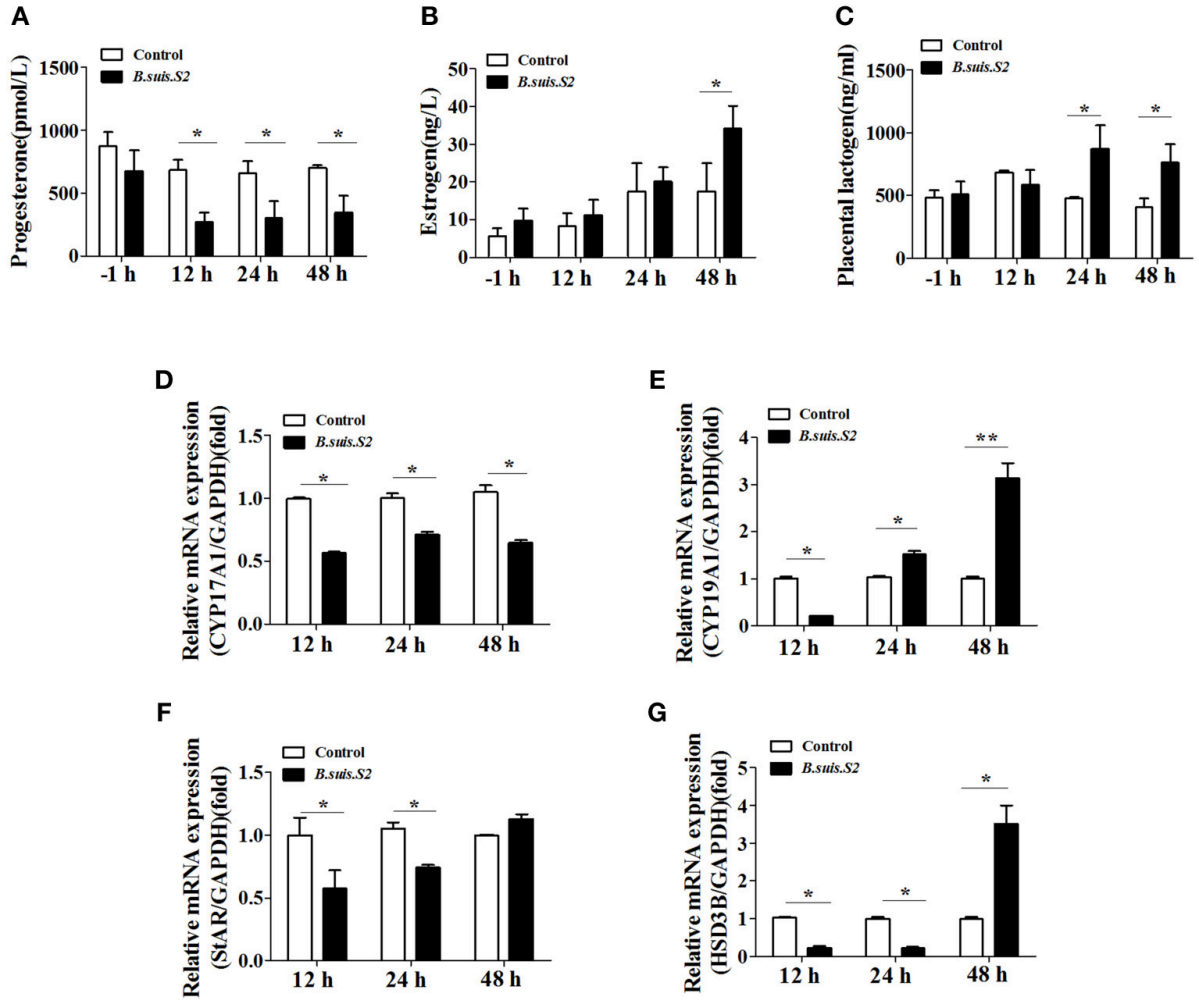
***B.suis.S2* infection disturbed the endocrine balance of GTCs**. GTC infection assays were performed as described in the Section Materials and Methods. Supernatants were collected at specific times (−1, 12, 24, and 48 h), and progesterone **(A)**, estrogen **(B)**, and prolactin **(C)** secretion was detected with ELISA kits according to the manufacturer's recommendations. Data represent the mean ± standard deviations from 5–6 independent experiments. ^*^P < 0.05 vs. non-infected GTCs. **(D–G)** Effects of *B.suis.S2* (MOI = 100:1) infection on the expression of genes encoding steroidogenic enzymes. Real-time PCR analysis revealed the expression of *CYP17A1* **(D)**, *CYP19A1* **(E)**, *StAR* **(F)**, and *HSD3B* **(G)** in *B.suis.S2*-infected GTCs at 12, 24, and 48 h. The data are corrected for expression of the housekeeping gene *GAPDH* (mean ±standard deviations from 5–6 independent experiments, ^*^P < 0.05, ^**^P < 0.01 vs. the non-infected GTCs).

The hormones secreted by trophoblast cells play important roles in reproductive functions (Filant and Spencer, 2014). *B.suis.S2* infection significantly inhibited the secretion of progesterone by GTCs at 12 h post-infection (Figure 7A), whereas estrogen secretion increased at 48 h (Figure 7B), and the secretion of placental lactogen increased after 24 h (Figure 7C). To further determine the mechanism for the disturbed hormone secretion observed with *B.suis.S2* infection, changes in the mRNA expression of genes encoding steroidogenic enzymes were detected by qRT-PCR. As shown in Figure 7D, the mRNA expression of *CYP17A1* decreased, in keeping with the decrease in P4 production in *B.suis.S2*-infected GTCs at 12 h post-infection. The expression of *CYP19A1* mRNA decreased at 12 h post-infection and increased after 24 h post-infection in *B.suis.S2*-infected GTCs (Figure 7E). The expression of *StAR* and *HSD3B* decreased before 24 h post-infection, which was roughly accompanied with decreases in *CYP17A1* mRNA expression and P4 production in *B.suis.S2*-infected cells. Thereafter, the expression of *HSD3B* increased, accompanied by increased *CYP19A1* mRNA expression and E2 production in *B.suis.S2*-infected cells at 48 h post-infection (Figures 7F,G).

Estrogen and progesterone target the endometrium through estrogen receptor α (EαR) and progesterone receptor (PR; Grewal et al., 2008). *B.suis.S2* infection in caprine endometrial epithelial cell lines (EECs) enhanced PR protein expression compared with the non-infected group at 24 and 48 h post-infection (Figures S5A,B), whereas EαR protein expression decreased at these time points (Figures S5A,C).

## Discussion

The results reported here indicated that *B.suis.S2* can rapidly infect and replicate in GTCs, significantly inducing growth retardation, increasing the percentage of apoptosis, and affecting endocrine functions under ER stress. Changes in UPR in GTCs affect the proliferation of *B.suis.S2* in these cells.

*B.suis.S2* can infect and replicate in GTCs; we observed an infection rate of ~99%, with growth accelerating at 12 h up to a maximum bacterial load after 24 h. This is consistent with a previous report that over 95% of macrophages became infected with RB51 at any tested MOI (Chen and He, 2009). Our results may explain why the vaccine based on this *Brucella* strain cannot be used in pregnant domestic animals.

Bacterial survival and replication inside host cells are critical for the establishment of chronic *Brucella* infection. Macrophages infected with rough *Brucella* strains undergo apoptotic cell death, whereas macrophages infected with smooth *Brucella* show inhibition of spontaneously occurring apoptosis (Gross et al., 2000; Tolomeo et al., 2003; He et al., 2006). The mechanisms by which smooth virulent *Brucella* inhibits apoptosis and promotes proliferation have been well demonstrated. Smooth virulent *Brucella* strains control or impact mitochondrial functions such as mitochondrial membrane transition and cytochrome c release (Chen and He, 2009), though these smooth strains do not induce caspase-2-dependent cell death. Caspase-2 also acts in concert with the mitochondrial and death receptor pathways of apoptosis (Sidi et al., 2008). In contrast, programmed cell death in macrophages infected with rough *Brucella* strains is partially due to a transition in mitochondrial permeability and caspase-2 activation. Recent studies have confirmed that smooth *Brucella* can dissociate into rough mutants that are cytotoxic to macrophages and conducive to the spread of *Brucella* (Pei et al., 2014). Therefore, we speculated that the inhibition and promotion of apoptosis occurs during the entire process of *Brucella* infection. In our studies, *B.suis.S2* infection induced caspase-8, -9, and CHOP protein expression for apoptosis in GTCs. Although the activation of caspase-8 and-9 was observed, inhibition of these caspases only slightly blocked *B. abortus*-induced PMN cell death (Barquero-Calvo et al., 2015). When ER stress is excessive and prolonged, CHOP levels increase to mediate ER stress-induced apoptosis (Groenendyk et al., 2010; Miyazaki et al., 2011). Therefore, we believe that ER stress-induced apoptosis plays an important role in *Brucella* infections.

Host UPR plays an absolutely critical role in supporting *Brucella* replication. GRP78 play essential roles in the biology and life cycles of viruses, bacteria, protozoa, and yeast cells, as well as higher eukaryotic cells. To guarantee persistent infection, Japanese encephalitis virus (JEV) and Hepatitis C Virus (HCV) prevent virus-induced apoptosis through constant up-regulation of GRP78 expression (Jiang et al., 2014; Lyoo et al., 2015). *B. abortus* takes advantage of VceC to interact with the ER chaperone GRP78 and localizes to the ER in HeLa cells (de Jong et al., 2013). *Streptomyces* inhibits GRP78 protein expression and induces cell death under ER stress (Hayakawa et al., 2014). Currently, Nexavar/Stivarga/Votrient is used to target GRP78 in the treatment of human malignancies, viral infections and bacterial diseases (Roberts et al., 2015). *Brucella* infection was recently suggested to induce UPR (Qin et al., 2008; de Jong et al., 2013; Smith et al., 2013), and the apparent effect of TUDCA in inducing GRP78 and CHOP expression is due to the greatly diminished numbers of bacteria (Smith et al., 2013). This finding is consistent with our result that inhibiting CHOP protein expression with 4-PBA increased the number of *B.suis.S2* CFUs in GTCs. In addition, enhancing CHOP protein expression with Tm inhibited the proliferation of *B.suis.S2* in GTCs. Enhancing expression of GRP78 and decreasing that of CHOP can also promote the proliferation of *B.suis.S2* in stably-transfected GRP78 over-expression GTC lines. Similarly, inhibiting GRP78 protein expression and increasing that of CHOP reduces *B.suis.S2* proliferation in stably-transfected GRP78 interference GTC lines. Thus, our study provides new evidence that increasing GRP78 expression promotes the proliferation of *B.suis.S2* in GTCs by inhibiting the apoptosis of *B.suis.S2*-infected GTCs.

Using insect cells and murine embryonic fibroblasts with IRE1 knockdown, Qin et al. (2008) demonstrated that bacterial replication is suppressed after UPR activation during *Brucella* infection. de Jong et al. (2013) and Yuki Taguchi et al. (2015) suggested that *B. abortus* infection activated the IRE1 pathway in HeLa cells, whereas Smith et al. (2013) showed that all three UPR pathways were induced after infection of murine macrophages with *B. melitensis*. In our study, we confirmed that *B.suis.S2* infection activated the IRE1 pathway and not the PERK and ATF6 pathways in GTCs. However, decreasing phosphoIRE1α and IRE1α expression with Irestatin 9389 in GTCs did not affect the number of *B.suis.S2* bacteria at 24 h post-infection. Our results are consistent with the report that IRE1 knockdown in BMDM did not affect the numbers of *B. abortus* cells at 24 h post-infection. However, host factor Yip1A mediated-IRE1 activation can be exploited by *B. abortus* to promote its replication in HeLa cells (Taguchi et al., 2015). This might reflect a difference between macrophages and epithelial cells with regard to different species of *Brucella*. IRE1 can regulate autophagic events independently from other ER-associated signaling molecules. Our results confirmed that autophagy was induced in *B.suis.S2*-infected GTCs, and decreasing LC3 protein expression with chloroquine decreased the number of *B.suis.S2* bacteria at 24 h post-infection. Taken together, ER stress induced in *Brucella* infection is a widespread phenomenon, and such changes in ER stress in either phagocytic or non-phagocytic cells can affect the proliferation of *Brucella* in different types of bacteria. However, our research confirmed that ER stress-apoptosis and ER-phagy signaling pathways, and not the ER stress pathway, play a decisive role in the process of *B.suis.S2* infection.

According to our results, GTC growth was retarded following *B.suis.S2* replication, but the implantation, migration, and invasion abilities of GTCs were not affected. These results are consistent with previous reports that only *B. melitensis* can interfere with trophoblast migration and invasion functions (Salcedo et al., 2013). Our study also proved that *B.suis.S2* infection simultaneously evoked ER stress and reduced the secretion of progesterone, though the secretion of estrogen and prolactin significantly increased. Our results are consistent with the report that *Brucella* bacteria preferentially replicate in placental trophoblasts during the middle and late stages of gestation after these cells actively secrete steroids (Gorvel and Moreno, 2002). In pregnancy, decreased progesterone or an increased estrogen to progesterone ratio lead to premature delivery, and progesterone increase and estrogen decrease are key events for triggering implantation in the secretory stage (Tabibzadeh, 1998). In addition, *B.suis.S2* infection disturbed PR and EαR protein expression under progesterone stimulation in EECs. Ultimately, not only the endocrine balance of trophoblast cells under normal growth conditions but also endometrial receptivity was defective, which may be a reason for the abortion and premature delivery induced by *Brucella* infection.

**Figure 8:**
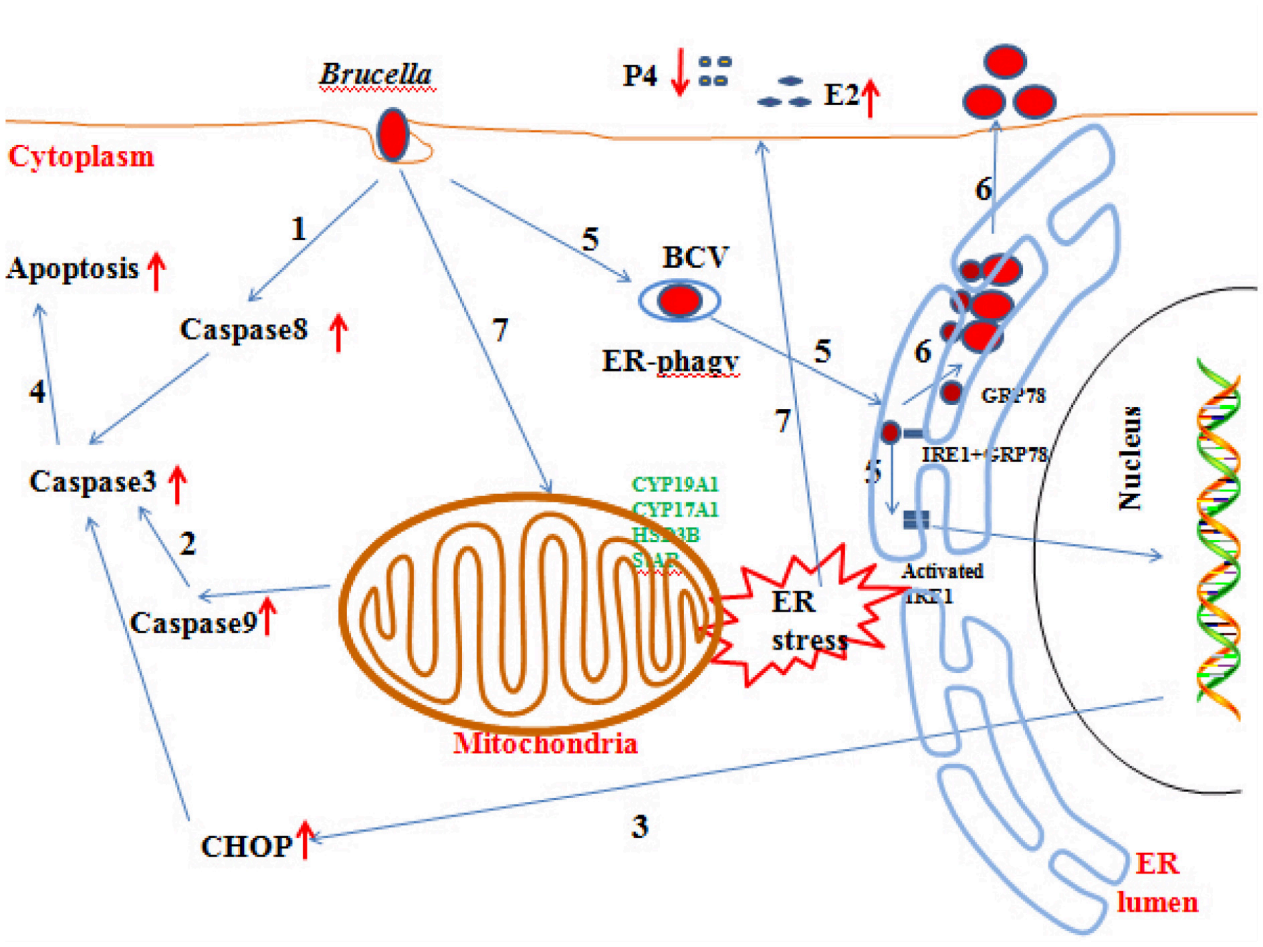
**Updated model of the response of GTCs to *B.suis.S2* infection. (1–5)** *Brucella* completes adhesion to and invasion into host cells. Survival and replication inside host cells is critical for the establishment of chronic *Brucella* infection. Virulent smooth *Brucella* inhibits programmed macrophage cell death and replicates inside macrophages (Gross et al., 2000; Tolomeo et al., 2003; He et al., 2006). Rough *Brucella* strains induce macrophage cell death (Chen and He, 2009). In our study, apoptosis and the apoptotic proteins caspase −8, −9, −3, and CHOP were induced in *B.suis.S2*-infected GTCs. **(6)** To date, scientists have elucidated the stealthy intracellular lifestyle of *Brucella* spp. in host cells, as described in the Section Introduction. IRE1a, an endoplasmic reticulum (ER) resident protein that plays a key role in regulating *Brucella* infection (Qin et al., 2008), is activated in *Brucella* infections. Under homeostatic conditions, BiP/GRP78 sequesters ER membrane proteins that function in UPR. *B.suis.S2* infection also induces ER stress in GTCs. Manipulating ER stress or GRP78 expression affects the proliferation of *B.suis.S2* in GTCs. However, manipulating IRE1α expression in GTCs does not affect the bacterial numbers of *B.suis.S2* at 24 h. **(7)** *Brucella* infection causes abortion and sterility in animals and debilitating disorders in humans. In our study, *B.suis.S2* disturbed the balance of P4 and E2 secretion under ER stress by mediating the expression of hormone-synthesis enzymes.

In this paper, we reveal the mechanism by which *Brucella* affects the host response and completes its proliferation. In addition, we also explore the mechanisms underlying the abortion induced by *Brucella* infection (Figure 8). Our findings may provide new insight for understanding the mechanisms involved in goat abortion caused by *Brucella* infections. Our work provides a starting point for exploring the function of ER stress and adds a new dimension to our understanding of the mechanism of abortion induced by *Brucella* infection. Our findings will also assist in the exploration of novel diagnosis and therapeutic strategies.

## Author contributions

XW and PL designed the experiments, interpreted the data, and wrote the article. XW and PL contributed equally to this work. XW performed the experiments with assistance and advice from YL, CX, YY, ZC, YD, DZ, and AW. AW and YJ revised the manuscript. All authors have read the manuscript and approved its submission.

### Conflict of interest statement

The authors declare that the research was conducted in the absence of any commercial or financial relationships that could be construed as a potential conflict of interest.

## Supplementary material

Figure S1 **Infection rates of *B.suis*.S2 in GTCs at 24 h**. *B.suis*.S2-mCherry (MOI = 100:1)-infected GTCs at 24 h (bar = 50 μm). The data shown are representative of 3–4 independent experiments.

Figure S2 **ER stress was induced by different concentrations of Tm and inhibited by different concentrations of 4-PBA in GTCs *in vitro*. (A)** GTCs were treated with different concentrations of Tm (0, 0.5, 1, 2 μg/ml) for 24 h and then evaluated by Western blotting. The data shown are representative of 5 independent experiments. **(B)** GTCs were treated with different concentrations of 4-PBA (0, 1, 10 μM) for 24 h in conjunction with 0.5 μg/ml Tm and then evaluated by Western blotting. The data shown are representative of 5 independent experiments. **(C)** Statistics of GTC apoptosis as determined by flow cytometry after 0.5 μg/mL Tm or 1 μM 4-PBA administration at 24 h. Data shown are representative of 4 independent experiments.

Figure S3 **IRE1α did not affect the proliferation of *B.suis.S2* at 24 h**. Confocal microscope images of IRE1α protein expression in *B.suis.S2*-infected GTCs with or without 10 μM Irestatin 9389 at 24 h post-infection. The data shown are representative of 3–4 independent experiments.

Figure S4 **ER-phagy involved in *B.suis.S2* infection. (A)** GTCs were infected with 100 MOI of *B.suis.S2*-mCherry for 12, 24, and 48 h, lysed and subjected to Western blot analysis to detect LC3 protein expression. The data shown are representative of 5 independent experiments. **(B)** GTCs were infected with 100 MOI of *B.suis.S2* with or without chloroquine (1.5, 4.5, or 10 μM) for 24 h, lysed and subjected to Western blot analysis to detect LC3 protein expression. The data shown are representative of 5 independent experiments. **(C)** Confocal microscope images of LC3 protein expression in *B.suis.S2*-infected GTCs with our without 10 μM chloroquine at 24 h post-infection. The data shown are representative of 3–4 independent experiments.

Figure S5 ***B.suis.S2* infection disturbed PR and EαR protein expression under progesterone stimulation in EECs**. Caprine endometrial epithelial cells (EECs) were cultured with complete DMEM/F12 medium containing P4 (10^−7^M, Sigma-Aldrich; Qin et al., 2015). *B.suis.S2* infection assays were performed as described in the Materials and Methods. EECs were collected at specific times (12, 24, and 48 h). **(A)** Protein expression levels of PR and EαR are shown for non-infected and *B.suis.S2*-infected cells at 12, 24, and 48 h. Data shown are representative of 4 independent experiments. The expression levels of PR **(B)** and EαR **(C)** were quantified using densitometry and normalized to the housekeeping protein β-actin. Data represent the mean ± standard deviations from 4 independent experiments, ^*^*P* < 0.05 versus non-infected GTCs).
