# Supplementary figures and images for: Brucella suis Vaccine Strain 2 Induces Endoplasmic Reticulum Stress that Affects Intracellular Replication in Goat Trophoblast Cells In vitro
Source: Front Cell Infect Microbiol. 2016 Feb 9;6:19. doi: 10.3389/fcimb.2016.00019 (PMC4746994; doi:10.3389/fcimb.2016.00019)

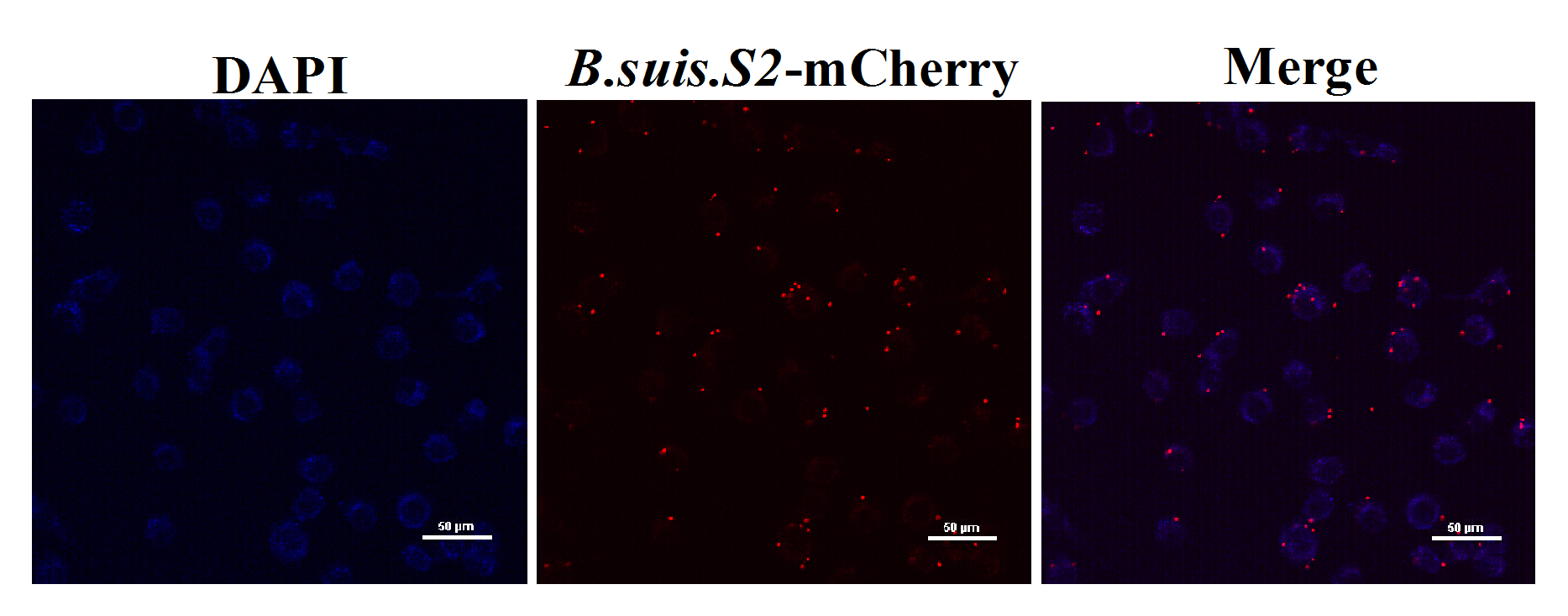

Supplement: Figure S1 — Infection rates of B.suis.S2 in GTCs at 24 h. B.suis.S2-mCherry (MOI = 100:1)-infected GTCs at 24 h (bar = 50 μm). The data shown are representative of 3–4 independent experiments. [file Image1.TIF]

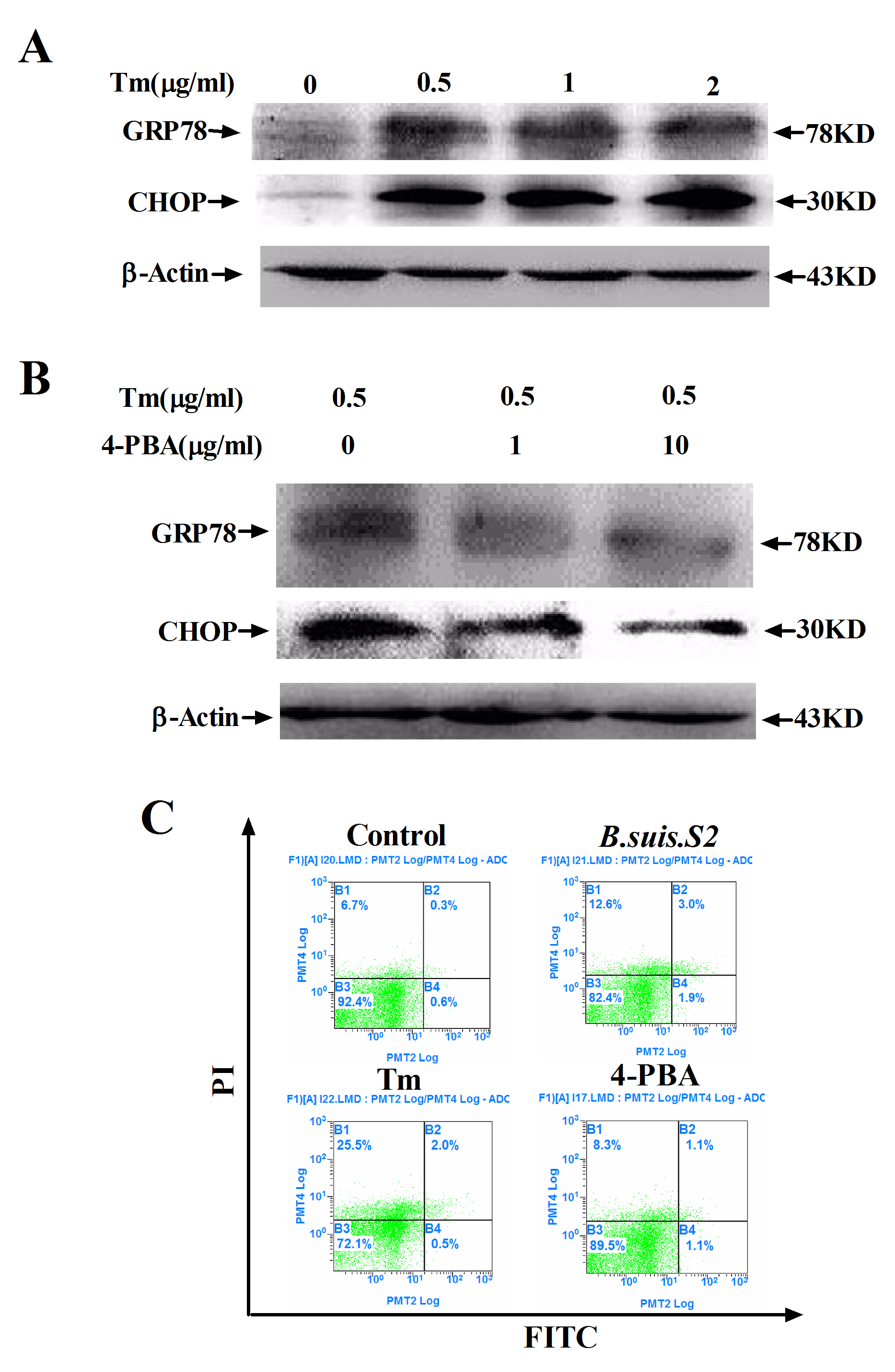

Supplement: Figure S2 — ER stress was induced by different concentrations of Tm and inhibited by different concentrations of 4-PBA in GTCs in vitro. (A) GTCs were treated with different concentrations of Tm (0, 0.5, 1, 2 μg/ml) for 24 h and then evaluated by Western blotting. The data shown are representative of 5 independent experiments. (B) GTCs were treated with different concentrations of 4-PBA (0, 1, 10 μM) for 24 h in conjunction with 0.5 μg/ml Tm and then evaluated by Western blotting. The data shown are representative of 5 independent experiments. (C) Statistics of GTC apoptosis as determined by flow cytometry after 0.5 μg/mL Tm or 1 μM 4-PBA administration at 24 h. Data shown are representative of 4 independent experiments. [file Image2.TIF]

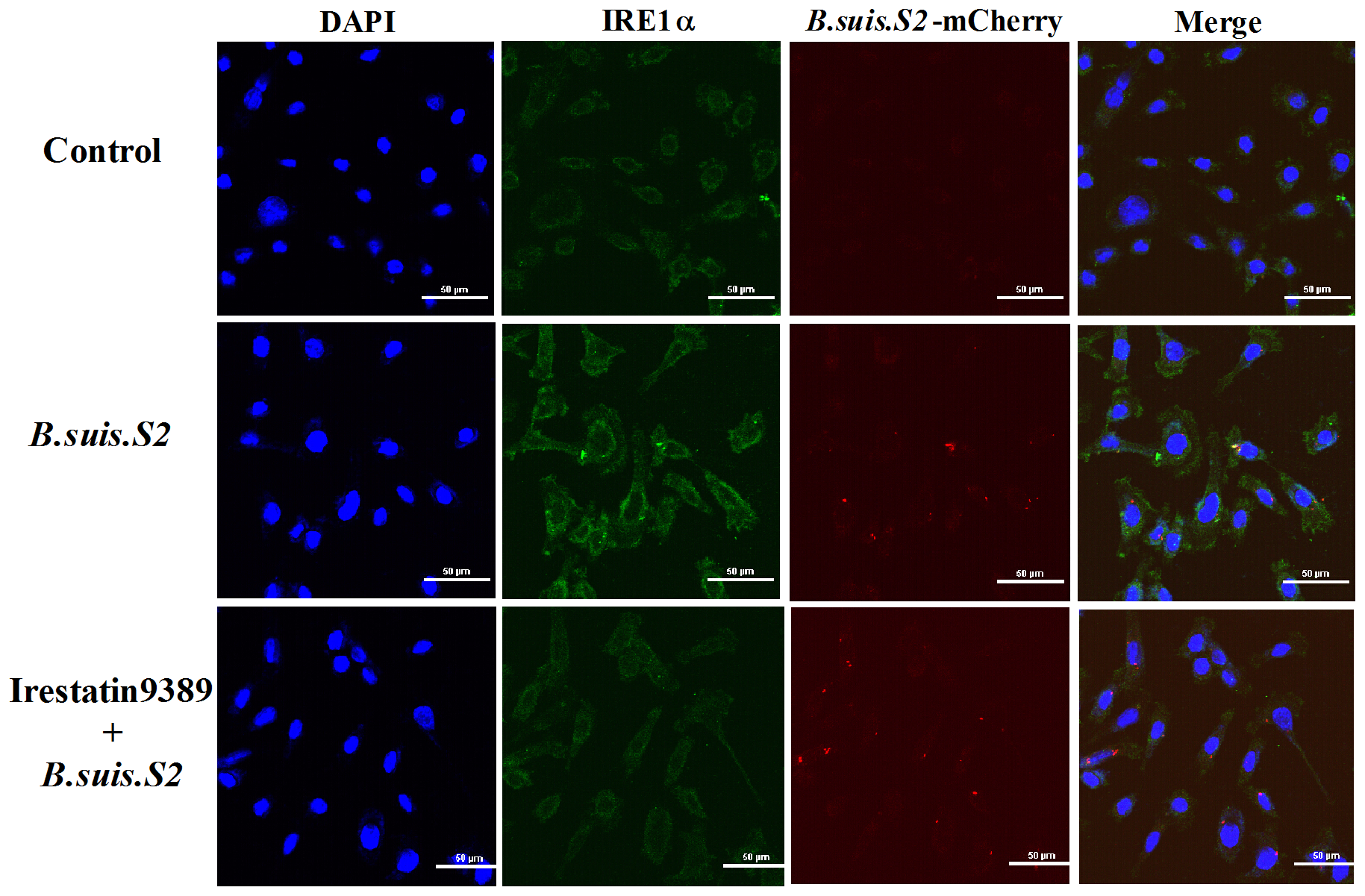

Supplement: Figure S3 — IRE1α did not affect the proliferation of B.suis.S2 at 24 h. Confocal microscope images of IRE1α protein expression in B.suis.S2-infected GTCs with or without 10 μM Irestatin 9389 at 24 h post-infection. The data shown are representative of 3–4 independent experiments. [file Image3.tif]

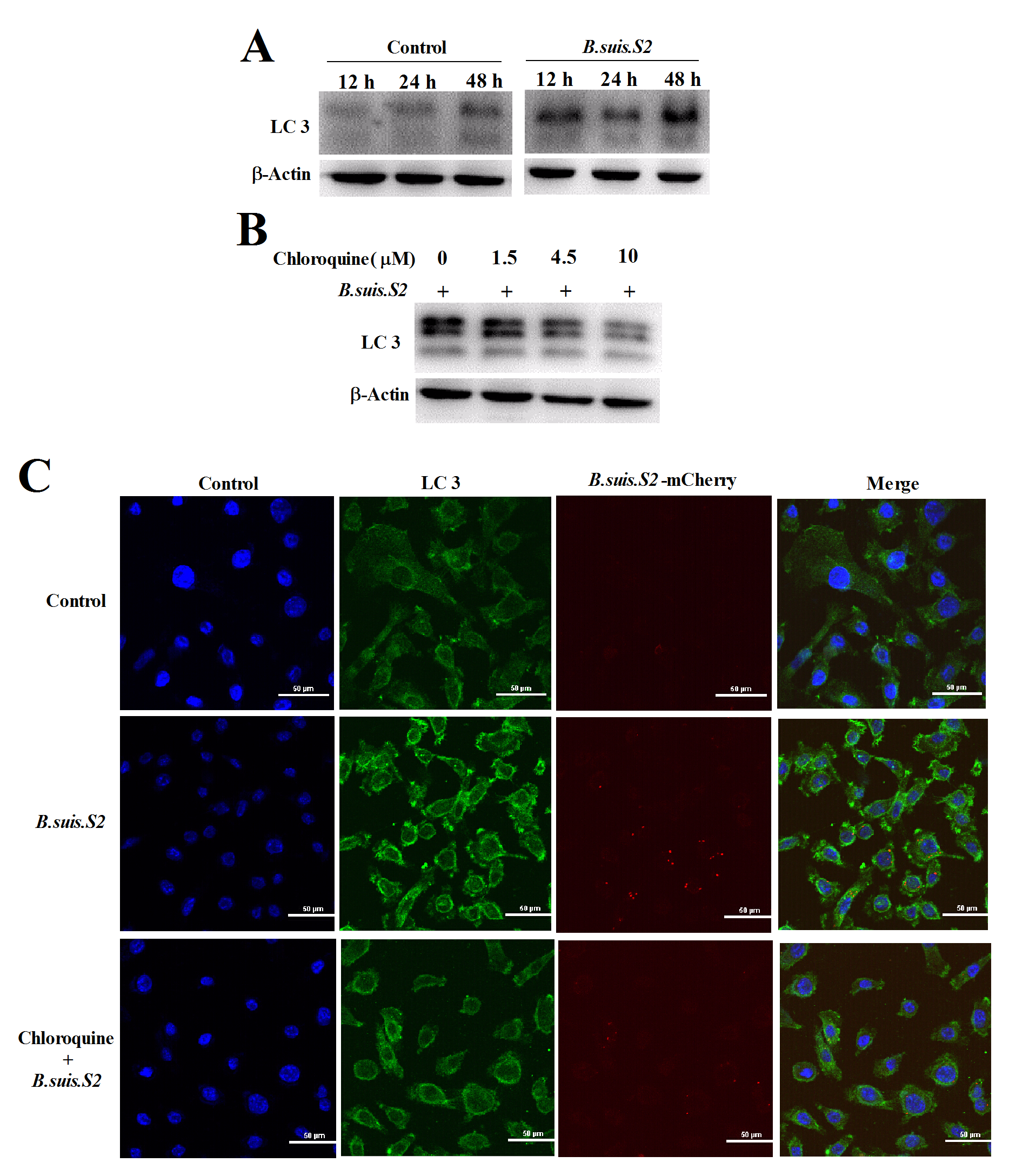

Supplement: Figure S4 — ER-phagy involved in B.suis.S2 infection. (A) GTCs were infected with 100 MOI of B.suis.S2-mCherry for 12, 24, and 48 h, lysed and subjected to Western blot analysis to detect LC3 protein expression. The data shown are representative of 5 independent experiments. (B) GTCs were infected with 100 MOI of B.suis.S2 with or without chloroquine (1.5, 4.5, or 10 μM) for 24 h, lysed and subjected to Western blot analysis to detect LC3 protein expression. The data shown are representative of 5 independent experiments. (C) Confocal microscope images of LC3 protein expression in B.suis.S2-infected GTCs with our without 10 μM chloroquine at 24 h post-infection. The data shown are representative of 3–4 independent experiments. [file Image4.TIF]

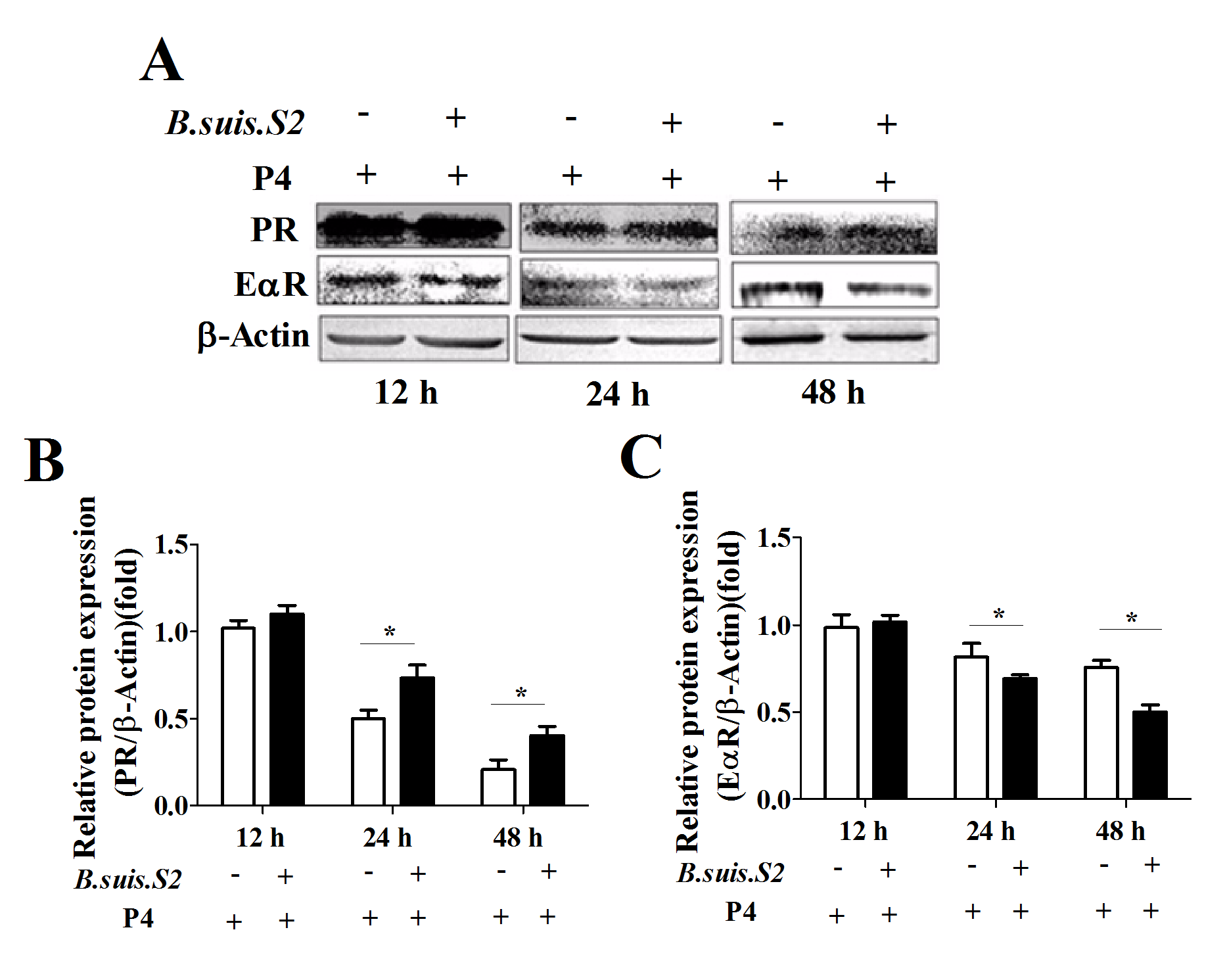

Supplement: Figure S5 — B.suis.S2 infection disturbed PR and EαR protein expression under progesterone stimulation in EECs. Caprine endometrial epithelial cells (EECs) were cultured with complete DMEM/F12 medium containing P4 (10−7M, Sigma-Aldrich; Qin et al., 2015). B.suis.S2 infection assays were performed as described in the Materials and Methods. EECs were collected at specific times (12, 24, and 48 h). (A) Protein expression levels of PR and EαR are shown for non-infected and B.suis.S2-infected cells at 12, 24, and 48 h. Data shown are representative of 4 independent experiments. The expression levels of PR (B) and EαR (C) were quantified using densitometry and normalized to the housekeeping protein β-actin. Data represent the mean ± standard deviations from 4 independent experiments, *P < 0.05 versus non-infected GTCs). [file Image5.TIF]
